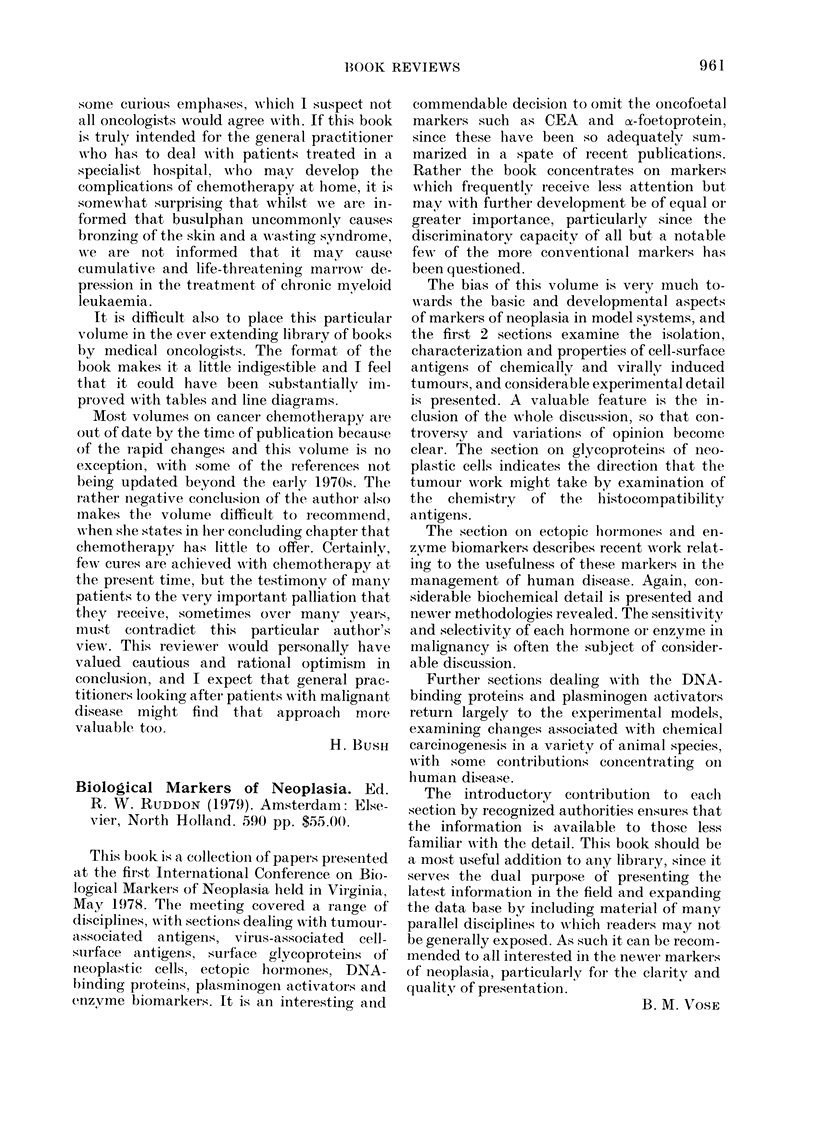# Biological Markers of Neoplasia

**Published:** 1979-12

**Authors:** B. M. Vose


**Biological Markers of Neoplasia.** Ed. R. W. Ruddon (1979). Amsterdam: Elsevier, North Holland. 590 pp. $55.00.

This book is a collection of papers presented at the first International Conference on Biological Markers of Neoplasia held in Virginia, May 1978. The meeting covered a range of disciplines, with sections dealing with tumour-associated antigens, virus-associated cell-surface antigens, surface glycoproteins of neoplastic cells, ectopic hormones, DNA-binding proteins, plasminogen activators and enzyme biomarkers. It is an interesting and commendable decision to omit the oncofoetal markers such as CEA and $\alpha$-foetoprotein, since these have been so adequately summarized in a spate of recent publications. Rather the book concentrates on markers which frequently receive less attention but may with further development be of equal or greater importance, particularly since the discriminatory capacity of all but a notable few of the more conventional markers has been questioned.

The bias of this volume is very much towards the basic and developmental aspects of markers of neoplasia in model systems, and the first 2 sections examine the isolation, characterization and properties of cell-surface antigens of chemically and virally induced tumours, and considerable experimental detail is presented. A valuable feature is the inclusion of the whole discussion, so that controversy and variations of opinion become clear. The section on glycoproteins of neoplastic cells indicates the direction that the tumour work might take by examination of the chemistry of the histocompatibility antigens.

The section on ectopic hormones and enzyme biomarkers describes recent work relating to the usefulness of these markers in the management of human disease. Again, considerable biochemical detail is presented and newer methodologies revealed. The sensitivity and selectivity of each hormone or enzyme in malignancy is often the subject of considerable discussion.

Further sections dealing with the DNA-binding proteins and plasminogen activators return largely to the experimental models, examining changes associated with chemical carcinogenesis in a variety of animal species, with some contributions concentrating on human disease.

The introductory contribution to each section by recognized authorities ensures that the information is available to those less familiar with the detail. This book should be a most useful addition to any library, since it serves the dual purpose of presenting the latest information in the field and expanding the data base by including material of many parallel disciplines to which readers may not be generally exposed. As such it can be recommended to all interested in the newer markers of neoplasia, particularly for the clarity and quality of presentation.

B. M. Vose